# Supporting systematic reviews using LDA-based document representations

**DOI:** 10.1186/s13643-015-0117-0

**Published:** 2015-11-26

**Authors:** Yuanhan Mo, Georgios Kontonatsios, Sophia Ananiadou

**Affiliations:** School of Computer Science, National Centre for Text Mining, The University of Manchester, Manchester, UK

**Keywords:** Topic model, Text mining, Machine learning, Systematic reviews

## Abstract

**Background:**

Identifying relevant studies for inclusion in a systematic review (i.e. screening) is a complex, laborious and expensive task. Recently, a number of studies has shown that the use of machine learning and text mining methods to automatically identify relevant studies has the potential to drastically decrease the workload involved in the screening phase. The vast majority of these machine learning methods exploit the same underlying principle, i.e. a study is modelled as a bag-of-words (BOW).

**Methods:**

We explore the use of topic modelling methods to derive a more informative representation of studies. We apply Latent Dirichlet allocation (LDA), an unsupervised topic modelling approach, to automatically identify topics in a collection of studies. We then represent each study as a distribution of LDA topics. Additionally, we enrich topics derived using LDA with multi-word terms identified by using an automatic term recognition (ATR) tool. For evaluation purposes, we carry out automatic identification of relevant studies using support vector machine (SVM)-based classifiers that employ both our novel topic-based representation and the BOW representation.

**Results:**

Our results show that the SVM classifier is able to identify a greater number of relevant studies when using the LDA representation than the BOW representation. These observations hold for two systematic reviews of the clinical domain and three reviews of the social science domain.

**Conclusions:**

A topic-based feature representation of documents outperforms the BOW representation when applied to the task of automatic citation screening. The proposed term-enriched topics are more informative and less ambiguous to systematic reviewers.

**Electronic supplementary material:**

The online version of this article (doi:10.1186/s13643-015-0117-0) contains supplementary material, which is available to authorized users.

## Background

The screening phase of systematic reviews aims to identify citations relevant to a research topic, according to a certain pre-defined protocol [1–4] known as the Population, the Intervention, the Comparator and the Outcome (PICO) framework. This framework seeks to identify the Population, the Intervention, the Comparator and the Outcome. This process is usually performed manually, which means that reviewers need to read thousands of citations during the screening phase, due to the rapid growth of the biomedical literature [5], making it an expensive and time-consuming process. According to Wallace et al. [6], an experienced reviewer is able to screen two abstracts per minute on average, with more complex abstracts taking longer. Moreover, a reviewer needs to identify all eligible studies (i.e. 95–100 % recall) [7, 8] in order to minimise publication bias. The number of relevant citations is usually significantly lower than the number of the irrelevant, which means that reviewers have to deal with an extremely imbalanced datasets. To overcome these limitations, methods such as machine learning, text mining [9, 10], text classification [11] and active learning [6, 12] have been used to partially automate this process, in order to reduce the workload, without sacrificing the quality of the reviews. Many approaches based on machine learning have shown to be helpful in reducing the workload of the screening phase [10]. The majority of reported methods exploit automatic or semi-automatic text classification to assist in the screening phase. Text classification is normally performed using the bag-of-words (BOW) model. The model assumes that the words in the documents are used as features for the classification, but their order is ignored. One of the problems of the BOW model is that the number of unique words that appear in a complete corpus (a collection of documents) can be extremely large; using such a large number of features can be problematic for certain algorithms. Thus, a more compact representation of documents is necessary to allow machine learning algorithms to perform more efficiently. In contrast to previous approaches that have used only BOW features, in this study, we systematically compare the two feature representations (Latent Dirichlet allocation (LDA) features and BOW features). Additionally, we investigate the effect of using different parameters (kernel functions) on the underlying classifier (i.e. support vector machine (SVM)).

### Topic analysis

Topic analysis is currently gaining popularity in both machine learning and text mining applications [13–16]. A topic model is normally defined as an approach for discovering the latent information in a corpus [17]. LDA [18] is an example of a probabilistic topic modelling technique [19], which assumes that a document covers a number of topics and each word in a document is sampled from the probability distributions with different parameters, so each word would be generated with a latent variable to indicate the distribution it comes from. By computing the extent to which each topic is represented in a document, the content of the document can be represented at a higher level than possible using the BOW approach, i.e. as a set of topics. The generative process of LDA follows the below steps to generate a document **w** in a corpus *D*, while Table 1 gives a list of all involved notation:

**Table 1:** Notation in LDA

*K*	Number of topics
$\vec {\alpha }$	Hyperparameter on document-topic distribution
$\vec {\beta }$	Hyperparameter on topics-word distribution
$\vec {\theta }_{m}$	A set of parameter vectors for generating a specific topic *z* in
	document *m*
**ϕ**	A set of parameter vectors for generating word *w*, according to *z*
*w*_*n*,*m*_	*n*th word in document *m*
*z*_*n*,*m*_	Topic indicator for *n*th word in document *m*

- Choose *K* topics **ϕ**∼Dir(*nβ*)
- Choose topics proportions $\vec {\theta }_{m} \sim \text {Dir}(\vec {\alpha })$
- For each word *w*_*n*_ in document *m*:

1. Choose a topic $z_{n,m} \sim \text {Multinomial}(\vec {\theta }_{m}) $
2. Choose a word *w*_*n*,*m*_ from $p(w_{n,m} |\vec {\phi }_{z_{n,m}}, \vec {\theta }_{m})$, a multinomial probability conditioned on the topic *z*_*n*_.

**Fig. 1:**
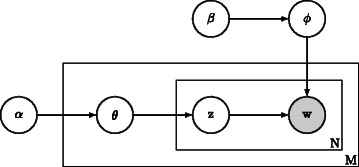
Latent Dirichlet allocation: a probability graphical model to describe how to construct a corpus. The *filled circle* denotes an observable variable

The hyperparameters $\vec {\alpha }$ and $\vec {\beta }$ are the parameters of the prior probability distributions which facilitate calculation. The hyperparameters are initialized as constant values. They may be considered as hidden variables which require estimation. The joint probability, i.e. the complete-data likelihood of a document, can be specified according to Fig. 1. The joint probability is the basis of many other derivations [20].

(1) $$ {\fontsize{7.7pt}{9.3pt}\selectfont{\begin{aligned} {}p\left(\vec{w}_{m}, \vec{z}_{m}, \vec{\theta}_{m}, \mathbf{\phi} ; \vec{\alpha}, \vec{\beta}\right)\! =\! \overbrace{\underbrace{\prod_{n=1}^{N_{m}} \!p\left(w_{n,m} | \vec{\phi}_{z_{n,m}}\right) p\left(z_{n,m} | \vec{\theta}\right)}_{\text{words in document}} \cdot p\left(\vec{\theta}_{m} | \vec{\alpha}\right)}^{\text{one document}} \cdot\underbrace{p\left(\mathbf{\phi}|\vec{\beta}\right)}_{\text{topics}} \end{aligned}}}  $$

Besides LDA, there are many other approaches for discovering abstract information from a corpus. Latent semantic analysis [21] makes use of singular value decomposition (SVD) to discover the semantic information in a corpus; SVD is a factorization of matrix which has many applications in statistics and signal processing. Unlike other topic models producing results, an approach [22] based on the anchor-word algorithm [23] provides an efficient and visual way for topic discovery. This method firstly reduces the dimensions of words co-occurrence matrix into two or three, then identify the convex hull of these words, which can be considered as a rubber band holding these words. The words at anchor points are considered as topics.

### Related work

Automatic text classification for systematic reviews has been investigated by Bekhuis et al. [24] who focussed on using supervised machine learning to assist with the screening phase. Octaviano et al. [25] combined two different features, i.e. content and citation relationship between the studies, to automate the selection phase as much as possible. Their strategy reduced workload by 58.2 %. Cohen et al. [26] compared different feature representations for supervised classifiers. They concluded that the best feature set used a combination of *n*-grams and Medical Subject Headings (MeSH) [27] features. Felizardo et al. developed a visual text mining tool that integrated many text mining functions for systemic reviews and evaluated the tool with 15 graduate students [28]. The results showed that the use of the tool is promising in terms of screening burden reduction. Fiszman et al. [29] combined symbolic semantic processing with statistical methods for selecting both relevant and high-quality citations. Frimza et al. [30] introduced a per-question classification method that uses an ensemble of classifiers that exploit the particular protocol used in creating the systematic review. Jonnalagadda et al. [31] described a semi-automatic system that requires human intervention. They successfully reduced the number of articles that needed to be reviewed by 6 to 30 % while maintaining a recall performance of 95 %. Matwin et al. [32] exploited a factorised complement naive Bayes classifier for reducing the workload of experts reviewing journal articles for building systematic reviews of drug class efficacy. The minimum and maximum workload reductions were 8.5 and 62.2 %, respectively, and the average over 15 topics was 33.5 %. Wallace et al. [12] showed that active learning has the potential to reduce the workload of the screening phase by 50 % on average. Cohen et al. [33] constructed a voting perceptron-based automated citation classification system which is able to reduce the number of articles that needs to be reviewed by more than 50 %. Bekhuis et al. [34] investigated the performance of different classifiers and feature sets in terms of their ability to reduce workload. The reduction was 46 % for SVMs and 35 % for complement naive Bayes classifiers with bag-of-words extracted from full citations. From a topic modelling perspective, Miwa et al. [8] firstly used LDA to reduce the burden of screening for systematic reviews using an active learning strategy. The strategy utilised the topics as another feature representation of documents when no manually assigned information such as MeSH terms is available. Moreover, the author used topic features for training ensemble classifiers. Similarly, Bekhuis et al. [35] investigated how the different feature selections, including topic features, affect the performance of classification.

## Methods

Results obtained by Miwa et al. [8] showed that LDA features can significantly reduce the workload involved in the screening phase of a systematic review. Building on previous approaches, we investigate how topic modelling can assist systematic reviews. By using topics generated by LDA as the input features for each document, we train a classifier and compare it with a classifier trained on the BOW representation. Technical terms extracted by the TerMine term extraction web service [36] were located in each document to allow them to be represented as a set of words and terms which would make topics more readable and eliminate ambiguity. The objectives of this paper are the following:

- To investigate whether LDA can be successfully applied to text classification in support of the screening phase in systematic reviews.
- To compare the performance of two methods for text classification: one based on LDA topics and the other based on the BOW model.
- To evaluate the impact of using different numbers of topics in topic-based classification.

### Experimental design

In order to carry out a systematic comparison of the two different approaches to text classification, our study is divided into two parts. Firstly, we evaluate the baseline approach, i.e. an SVM using BOW features. This SVM classifier is created using LIBSVM [37]. The second part of the experiment involves applying LDA for modelling topic distribution in the datasets, followed by the training of an SVM-based classifier using the topic distribution as features. Documents in the dataset are randomly and evenly spilt into training and test sets, keeping the ratio between relevant and irrelevant documents in each set the same as the ratio in the entire dataset. Henceforth, in this article, the documents relevant to a topic (i.e. positively labelled instances) are referred to as “relevant instances”. BOW features are weighted by term frequency/inverse document frequency (TF-IDF) as a baseline. The topic-based approach applies LDA to produce a topic distribution for each document. We used Gensim [38], an implementation of LDA in Python, to predict the topic distribution for each document. The topic distributions are utilised for both training and testing the classifier and evaluating the results. Other modelling strategies and classifiers (e.g. *k*-nearest neighbours) were also explored. However, since they failed to obtain robust results, we do not present further details.

To evaluate the classifiers, the standard metrics of precision, recall, *F*-score, accuracy, area under the receiver operating characteristic curve (ROC) and precision-recall curve (PRC). However, in our case, accuracy was found not to be a suitable indicator of an effective performance, due to the significant imbalance between relevant and irrelevant instances in the dataset; this ratio is 1:9 approximately for each corpus (Table 2) which will be introduced later. Based upon this ratio, weights are added to every training instance in order to reduce the influence caused by imbalanced data [39]. In evaluating classification performance, we place a particular emphasis on recall since, as explained above, high recall is vital to achieve inclusiveness, which is considered to be such an important factor in the perceived validity of a systematic review.

**Table 2:** Corpus information

	Positive instances	Total instances	Ratio	Feature used	Type
Youth development	1440	14,538	0.099	Title + abstract	Social science
Cigarette packaging	132	3156	0.041	Title + abstract	Social science
COPD	196	1606	0.122	Title + abstract	Clinical trial
Cooking skill	197	9439	0.021	Text	Social science
Proton beam	243	4751	0.051	Title + abstract	Clinical trials

Since most of our corpora are domain-specific, non-compositional multi-word terms may lose their original meaning if we split such terms into constituent words and ignore word order and grammatical relations. Thus, multi-word terms are automatically extracted using TerMine, which is a tool designed to discover multi-word terms by ranking candidate terms from a part-of-speech (POS) tagged corpus according to C-value [36]. Candidate terms are identified and scored via POS filters (e.g. adjective*noun+). A subset of these terms is extracted by defining a threshold for the C-value. TerMine makes use of both linguistic and statistical information in order to identify technical terms in a given corpus with the maximum accuracy. There are some other topic models that attempt to present multi-word expressions in topics. For example, the LDA collocation model [40] introduced a new latent variable to indicate if a word and its immediate neighbour can constitute a collocation. Unlike the methods mentioned, the advantage of TerMine is that it is applied independently of the topic modelling process. Thus, once it has been used to locate terms in a corpus, different topic models can be applied, without having to re-extract the terms each time the parameters of the topic model are changed. It is also important to note that long terms may have other shorter terms nested within them. Such nested terms may also be identified by TerMine. For example, “logistic regression model” contains the terms “logistic regression” and “regression model”. However, there is no doubt that the original term “logistic regression model” is more informative. Thus, our strategy to locate the terms is that the longer terms are given higher priority to be matched and our maximum length for a term is four tokens.

As for parameter tuning, all the experiments have been performed with default parameters for classifiers and symmetry hyperparameters for LDA, which means that every topic will be sampled with equal probability.

## Results and discussion

We performed our experiments using five datasets corresponding to completed reviews, in domains of social science and clinical trials. These reviews constitute the “gold standard” data, in that for each domain, they include expert judgements about which documents are relevant or irrelevant to the study in question. The datasets were used as the basis for the intrinsic evaluation of the different text classification methods. Our conclusions are supported by the Friedman test (Table 3) which is a nonparametric test that measure how different three or more matched or paired groups are based on ranking. Given that the methods we applied produced roughly comparable patterns of performance across each of the five different datasets, we report here only on the results for one of the corpora. However, the specific results achieved for the other corpora are included as supplementary material (Additional file 1).

**Table 3:** Friedman test for five datasets on different kernel functions and documents representation

		Linear			RBF			POLY	
	BOW	TPC	TE	BOW	TPC	TC	BOW	TPC	TE
Precision									
Mean rank	2.90	2.00	1.10	1.00	2.50	2.50	1.2	2.6	2.2
*P* =	0.0001			0.00196			0.001501		
Recall									
Mean rank	1.00	2.60	2.40	1.00	2.40	2.60	1.20	2.40	2.40
*P* =	0.00332			0.0256			0.008977		
*F*-score									
Mean rank	2.60	2.10	1.30	1.00	2.60	2.40	1.20	2.60	2.20
*P* =	0.08977			0.00332			0.01501		
ROC									
Mean rank	3.00	1.80	1.20	1.00	2.60	2.40	1.00	2.60	2.40
*P* =	0.00066			0.00332			0.00332		
PRC									
Mean rank	2.80	2.00	1.20	1.00	2.70	2.30	1.00	2.60	2.40
*P* =	0.0168			0.0008			0.84935		

### Dataset

We applied the models to three datasets provided by the Evidence Policy and Practice Information and Coordinating Center (EPPI-center) [41] and two datasets previously presented in Wallace et al. [6]. These labelled corpora include reviews ranging from clinical trials to reviews in the domain of social science. The datasets correspond specifically to cigarette packaging, youth development, cooking skills, chronic obstructive pulmonary disease (COPD), proton beam and hygiene behaviour. Each corpus contains a large number of documents and, as mentioned above, there is an extremely low proportion of relevant documents in each case. For example, the youth development corpus contains a total of 14,538 documents, only 1440 of which are relevant to the study. Meanwhile, the cigarette packaging subset contains 3156 documents in total, with 132 having been marked as relevant. Documents in the datasets were firstly prepared for automatic classification using a series of pre-processing steps consisting of stop-word removal, conversion of words to lower case and removal of punctuation, digits and the words that appear only once. Finally, word counts were computed and saved in a tab-delimited format (SVMlight format), for subsequent utilisation by the SVM classifiers. Meanwhile, TerMine was used to identify multi-word terms in each document, as the basis for characterising their content. Preliminary experiments indicated that only using multi-word terms to characterise documents may not be sufficient since, in certain documents, the number of such terms could be small or zero. Accordingly, words and terms were retained as features for an independent experiment.

### BOW-based classification

**Fig. 2:**
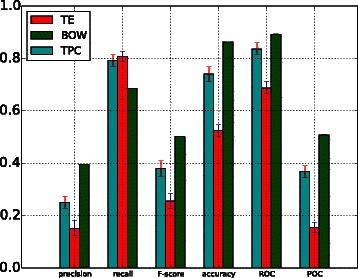
Linear kernel function. Comparison between the performance of BOW-based, topic distribution-based and term-enriched topic classifiers trained using a linear kernel function

Table 4 shows the performance of the SVM classifiers trained with TF-IDF features when applied to all corpora. Due to the imbalance between relevant and irrelevant instances in the dataset, each positive instance was assigned a weight, as mentioned above. Default values for SVM training parameters were used (i.e. no parameter tuning was carried out), although three different types of kernel functions were investigated, i.e. linear, radial basis function (RBF) and polynomial (POLY). Unlike the linear kernel that aims to find a unique hyperplane between positive and negative instances, RBF and POLY can capture more complex distinctions between classes than the linear kernel. As illustrated in Fig. 2, the BOW-based classification achieves the best performance when the linear kernel function is used. However, it is necessary to recall that the ratio of positively (i.e. relevant) to negatively (i.e. irrelevant) labelled instances is approximately 1:9 in our corpora. Hence, even if a classifier labels all test samples as irrelevant instances, a very-high accuracy will still be obtained. However, for systematic reviews, it is most important to retrieve the highest possible number of relevant documents; recall is a much better indicator of performance than accuracy. Secondly, both the RBF and polynomial kernel functions obtained zero for precision, recall and *F*_1_-score. This can be attributed to the imbalanced nature of the corpora [42]. Additionally, the BOW representation produces a high dimensional space (given the large number of unique words in the corpora). In this high dimensional space, the two non-linear kernels (RFB and POLY) yield a very low performance.

**Table 4:** Evaluation on all corpora of SVM classifiers trained with TF-IDF features

	Precision	Recall	*F*_1_-score	Accuracy	ROC	PRC
Youth development						
Linear	0.394799	0.686301	0.50125	0.8628422	0.891629	0.508361
RBF	0.0	0.0	0.0	0.89957353	0.13187	0.055498
POLY	0.0	0.0	0.0	0.8995735	0.15324	0.054825
Cigarette packaging						
Linear	0.3679999	0.7076923	0.48421052	0.937896	0.939295	0.477252
RBF	0.0	0.0	0.0	0.9588086	0.06347	0.021359
POLY	0.0	0.0	0.0	0.9588086	0.082638	0.021496
Cooking skill						
Linear	0.366666	0.482456	0.416666	0.967365	0.922862	0.328018
RBF	0.0	0.0	0.0	0.9758	0.07937	0.012568
POLY	0.0	0.0	0.0	0.97584233	0.51207	0.500
COPD						
Linear	0.59523	0.773195	0.67264	0.909	0.927631	0.720464
RBF	0.0	0.0	0.0	0.8792	0.066893	0.064489
POLY	0.0	0.0	0.0	0.8792	0.1139	0.067315
Proton beam						
Linear	0.0574	0.07874	0.0664451	0.881734	0.562028	0.063233
RBF	0.0	0.0	0.0	0.9465	0.442163	0.048747
POLY	0.0	0.0	0.0	0.9465	0.482718	0.05424

*RBF* radial basis function kernel, *POLY* polynomial kernel

### Topic-based classification

Topic-based classification was undertaken by firstly analysing and predicting the topic distribution for each document and then classifying the documents using topics as features. During the phase of training the model, the topic assigned to each word in a document can be considered as a hidden variable, this problem can be solved by using approximation methods such as Monte Carlo Markov chain (MCMC) or variational inference. However, these methods are sensitive to initial parameter settings which are usually set randomly before the first iteration. Consequently, the results could fluctuate within a certain range. The results produced by topic-based classification are all average results. However, our results show that topic distribution is an ideal replacement for the traditional BOW features. Besides other advantages, the most obvious advantage of which is to reduce the dimensions of features for representing a document. Experimental settings were identical in the evaluation of the two sets of classifiers, except for the features being topic distributions in one case and BOW in the other. The optimal LDA model was derived through experimentation with differing numbers of topics (which can also be referred to as “topic density”). In the experiments performed, several values for this parameter were explored.

Table 5 shows the results of the evaluation of SVM models trained with topic distribution features using linear, RBF and POLY kernel functions, respectively. We show how the performance varies according to different topic density values for the LDA model. These values were varied from 2 to 100 (inclusive), in increments of 10, and from 100 to 500 in increments of 100 approximately. Generally, each topic density would correspond to a certain size of corpus and vocabulary. Empirically, the larger the size of the corpora and vocabulary, the greater the number of topics that is needed to accurately represent their contents, and vice versa. Tables 6 and 7 show two samples of sets of words and/or terms that are representative of a topic in the same corpus (youth development). Term-enriched (TE) topics include multi-word terms identified by TerMine as well as single words, whilst ordinary topics consist only of single words. From the tables, it can be clearly seen that term-enriched topics are more distinctive and readable than single-word topics. As the classification performance was similar to the single-word topic-based classification, a table like Table 5 will not be presented here. However, a comparison of the classification performance for the three approaches, i.e. BOW-based, topic-based and TE-topic-based will be presented in the next section.

**Table 5:** Evaluation on the youth development data set of SVM classifiers trained with topic features

Topic density^a^	Precision	Recall	*F*_1_-score	Accuracy	ROC	PRC
Linear						
2	0.15659	0.76767	0.28124	0.55268	0.685362	0.16959
5	0.16389	0.76986	0.28025	0.61253	0.7485	0.215196
10	0.21661	0.775616	0.334683	0.706321	0.782012	0.239912
20	0.22839	0.7767123	0.31605	0.66244	0.77806	0.276948
30	0.235857	0.772465	0.357992	0.732592	0.816773	0.288951
40	0.239157	0.7730136	0.36417129	0.73246	0.820795	0.320774
50	0.232558	0.7671232	0.35691523	0.72242	0.818062	0.371289
60	0.2494141	0.7771232	0.35320	0.717881	0.811705	0.338449
70	0.283706	0.7719178	0.407421	0.789108	0.841333	0.342748
80	0.27956	0.782191	0.3548788	0.782191	0.84254	0.359227
90	0.28068	0.77479	0.376366	0.748830	0.832757	0.345683
100	0.28137	0.786575	0.376121	0.751306	0.831486	0.358541
150	0.29082	0.79178	0.379514	0.740751	0.836747	0.367825
200	0.2949	*0.79123*	0.423232	0.77078	*0.850254*	*0.40842*
300	*0.3224*	0.72054	*0.4558*	0.9588086	0.847479	0.389575
500	0.3059	0.7082	0.4272	0.80935	0.844137	0.39549
RBF						
2	0.151121	0.812328	0.2548345	0.5229	0.694288	0.168685
5	0.159186	*0.826027*	0.26693	0.54436	0.719956	0.194878
10	0.189766	0.802739	0.306966	0.635988	0.775362	0.201232
20	0.19948	0.7452	0.314723	0.674095	0.774715	0.253942
30	0.257261	0.679452	0.373213	0.77082	0.816608	0.312387
40	0.264912	0.6205	0.37131	0.78896	0.799286	0.301266
50	0.246453	0.641098	0.356534	0.767093	0.779354	0.250867
60	0.23598	0.57671	0.33492	0.76998	0.77882	0.250866
70	0.255531	0.49041	0.33532	0.80533	0.773743	0.237212
80	0.39523	0.39041	*0.38255*	0.873435	0.806185	0.318034
90	*0.4092*	0.219178	0.285459	0.889806	0.801959	0.312336
100	0.368421	0.019178	0.03645	0.898197	*0.817434*	*0.319278*
150	0	0	0	0.877579	0.812314	0.297883
POLY						
2	0.153	*0.82602*	*0.25818*	0.5233	0.70262	*0.170105*
5	*0.17164*	0.14315	0.156498	0.843513	*0.70445*	0.166452
10	0	0	0	0.899574	0.285556	0.06007

Items in italics refer to the highest scores obtained in a column

^a^Results are reported according to different values of the topic density

**Table 6:** Term-enriched topics

Topic 1	Topic 2	Topic 3
School	*Teen birth rates*	*Program activity*
Plains	School	Murders
Murders	Weakly	Educare
*Cultural tradition*	Corresponds	Projected
*Gang membership*	Ngos	*Multidimensional index*
*Juvenile delinquency prevention program*	Chile	*Program activity*
Immigration	*Latino culture*	*Fast track*
Educare	Wore	*Socio-economic circumstance*
Recollections	*Nonneglected children*	*Nonneglected children*
*Program activity*	Skillful	Hopkins
Topic 4	Topic 5	Topic 6
*Medical students*	*Mental health worker*	Shrinking
*Program evaluators*	Skilful	Murders
Nepal	Cortical	*Social disorganization*
Coverform	Trauma	*Gang membership*
Selfconfidence	Papel	Herd
Suicidality	*Longitudinal designs*	*Medical student*
*Risk protective*	Commentators	Kofi
Reasoned	Jugend	Ordered
Discontinue	*Original abstractamendedcd coden chdeaw*	*Outdoor adventure program*
Breed	*Cultural system*	Projected

Items in italics refer to multi-word terms

**Table 7:** Ordinary topics

Topic 1	Topic 2	Topic 3
Forged	Bosnian	Horizons
School	Acculturationrelated	Pascd
Educare	Revitalization	Steps
Nonconcordant	Chipce	Healthier
Nonfarmers	Api	Wore
Eightythree	Unavailability	Fibrosis
Mdma	Paradigmatic	Eurocentric
Privatized	Individualist	Justified
Chile	Phonics	Noncollege
Discontinue	Fulfils	Correspond
Topic 4	Topic 5	Topic 6
Cindy	Abortions	Infectious
Completions	Mediocre	Adequate
Phonics	Estimates	Memethods
Psychotic	Daysweek	Phonics
Mdma	Cubic	Personalized
Healthier	Midwestern	Thyroxine
Otherfoucsed	Preceded	Apparent
Fibrosis	Interventional	Twentieth
Suzanne	Selfsilencing	Outdoor
School	Evenings	Verbally

### Comparison of approaches

**Fig. 3:**
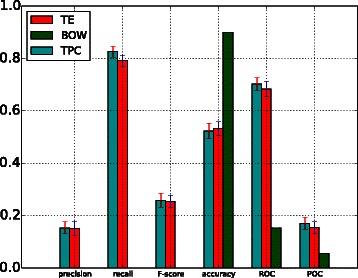
RBF kernel function. Comparison between the performance of BOW-based, topic distribution-based and term-enriched topic classifiers trained using an RBF kernel function

**Fig. 4:**
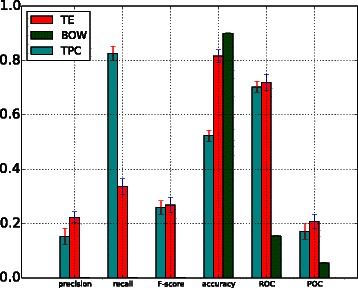
POLY kernel function. Comparison between the performance of BOW-based, topic distribution-based and term-enriched topic classifiers trained using a POLY kernel function

**Fig. 5:**
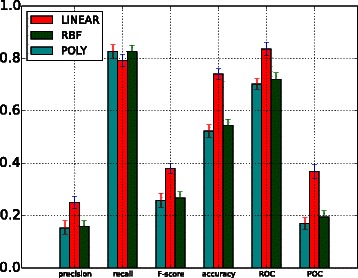
Different kernel functions. Comparison between the performance of linear, RBF and POLY kernel functions using topic feature

**Fig. 6:**
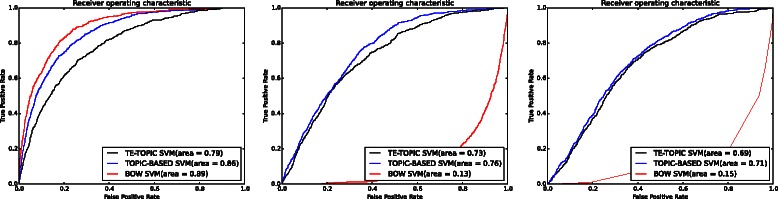
Receiver operation curve: each figure was produced using a kernel function. *Left*: linear kernel function. *Middle*: RBF kernel function. *Right*: POLY kernel function

**Fig. 7:**
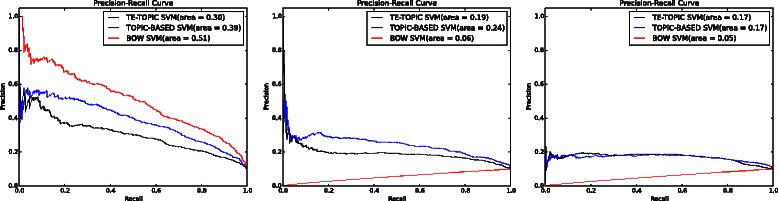
Precision-recall curve: each figure was produced using a kernel function. *Left*: linear kernel function. *Middle*: RBF kernel function. *Right*: POLY kernel function

A comparison of the performance of the BOW-based model (BOW in legend) against the performance of models trained with topic-based model (TPC) and term enriched-topic model (TE) is presented in this section. According to the results of using a linear function for model training (Fig. 2), models based on topic and TE-topic distribution features yield lower precision, *F*-score, ROC and PRC but obtain higher recall. For this comparison, the best performing topic-based model (with topic density set to 150 for youth development corpus) was used. It can be observed from Fig. 2 that the BOW-based model outperforms the topic- and TE-topic based one in terms of all metrics except for recall. Figures 3 and 4 illustrate the results of using RBF and POLY kernel functions, respectively, in training BOW, topic-based models and TE-topic-based model on the youth development corpus. It can be observed that employing these kernels, the SVM models trained with topic and TE-topic distributions outperform those trained with BOW features by a large margin. Another observation is that training using RBF and POLY kernel functions significantly degraded the performance of BOW-based models. Using RBF and POLY kernel functions, the BOW-based classifiers perform poorly, with zero in precision, recall and *F*-score. As noted earlier, high accuracy is not a good basis for judging performance due to the imbalance between positive and negative instances, i.e. even if a classifier labels every document as a negative sample, accuracy will still be around 90 %. Figure 5 gives the comparison of different kernel functions using topic features on the youth development corpus, indicating that taking all measures into account, a linear kernel function gave the best overall performance, achieving the highest score in every metric other than recall. However, both RBF and POLY kernel functions outperformed linear, albeit by only 4 %, on the recall measure, which we have identified as highly pertinent to the systematic review use-case. We used a generic list of kernel functions ranked from high to low in terms of recall for topic-based and TE-topic-based feature in Table 8: POLY >*RBF*>LINEAR. For a ranked list of feature types in terms of recall, it is: TPC >*TE*>BOW. Additionally, Figs. 6 and 7 show precision-recall and ROC curves achieved by the models.

**Table 8:** The performance of all corpus with different feature selection and kernel functions

	BOW						TPC						TE					
	P	R	F	A	ROC	PRC	P	R	F	A	ROC	PRC	P	R	F	A	ROC	PRC
Linear																		
Youth development	0.394	0.686	0.501	0.862	0.891	0.508	0.249	0.791	0.379	0.740	0.836	0.367	0.151	0.808	0.255	0.525	0.688	0.154
Cigarette packaging	0.367	0.707	0.484	0.937	0.939	0.477	0.062	0.969	0.011	0.397	0.750	0.070	0.062	0.953	0.011	0.411	0.662	0.066
Cooking skill	0.366	0.482	0.416	0.967	0.922	0.328	0.038	0.947	0.073	0.422	0.701	0.038	0.032	0.921	0.061	0.326	0.717	0.051
COPD	0.595	0.773	0.672	0.909	0.927	0.720	0.418	0.876	0.566	0.838	0.893	0.557	0.184	0.907	0.306	0.504	0.714	0.202
Proton beam	0.057	0.078	0.066	0.881	0.562	0.063	0.057	0.606	0.105	0.452	0.547	0.068	0.054	0.551	0.098	0.460	0.479	0.051
RBF																		
Youth development	0.0	0.0	0.0	0.899	0.131	0.055	0.159	0.826	0.266	0.544	0.719	0.194	0.145	0.809	0.246	0.501	0.679	0.156
Cigarette packaging	0.0	0.0	0.0	0.958	0.063	0.021	0.0550	0.986	0.104	0.293	0.729	0.094	0.063	0.923	0.118	0.435	0.693	0.082
Cooking skill	0.0	0.0	0.0	0.9758	0.079	0.012	0.032	0.894	0.063	0.363	0.651	0.033	0.032	0.938	0.061	0.313	0.660	0.033
COPD	0.0	0.0	0.0	0.879	0.066	0.064	0.3577	0.804	0.495	0.801	0.882	0.506	0.169	0.958	0.287	0.427	0.702	0.189
Proton beam	0.0	0.0	0.0	0.9465	0.442	0.048	0.053	0.716	0.099	0.305	0.474	0.049	0.055	0.724	0.103	0.330	0.511	0.053
POLY																		
Youth development	0.0	0.0	0.0	0.899	0.153	0.054	0.153	0.826	0.258	0.523	0.702	0.170	0.151	0.791	0.253	0.532	0.683	0.153
Cigarette packaging	0.0	0.0	0.0	0.958	0.082	0.021	0.059	0.986	0.112	0.349	0.660	0.070	0.061	1.000	0.115	0.366	0.664	0.067
Cooking skill	0.0	0.0	0.0	0.975	0.512	0.500	0.037	0.938	0.072	0.418	0.703	0.039	0.031	0.903	0.061	0.332	0.655	0.043
COPD	0.0	0.0	0.0	0.8792	0.113	0.067	0.262	0.824	0.398	0.698	0.799	0.278	0.195	0.896	0.320	0.540	0.715	0.196
Proton beam	0.0	0.0	0.0	0.9465	0.482	0.054	0.0	0.0	0.0	0.946	0.483	0.050	0.0	0.0	0.0	0.946	0.489	0.052

*BOW* bag-of-word feature, *TPC* topic feature, *TE* term-enriched topic feature

## Conclusions

Our experiments demonstrated that the performance of BOW SVM with linear kernel function has produced the most robust results achieving the highest values in almost every metric, except for recall. But on any systematic reviews classification task, poor performance in recall needs to be addressed. The BOW model yielded a poor performance with RBF and POLY kernel functions due to the data imbalance and dimensionality issue. Topic-based classification significantly addresses this problem by dramatically reducing the dimensionality of the representation of a document (topic feature). The topic-based classifier yielded a higher recall, which means more relevant documents will be identified. Moreover, the topic features enable the classifier to work with RBF and POLY kernels and produce better recall comparing with a linear kernel. The same patterns were observed in all corpora, although there is only one example presented in this article.

As future work, we will further investigate the generalisability of the model to diverse domains. Moreover, we plan to explore different machine learning and text mining techniques that can be used to support systematic reviews such as paragraph vectors and active learning. Also, further experiments will be performed in a more realistic situation. For example, whether topics could help reviewers’ decision in “live” systematic review would be an interesting research area in the future. An intuitive image of TE topics has been made in this article. For public health reviews where topics are multidimensional, the presence of diverse multi-word terms in a dataset can be an important element that affects the performance of classifiers. But TE topics have the potential to deal with these difficulties. Further investigation on TE topics will be performed, which would benefit reviewers and help them to understand topics more easily compared to ordinary topics.

## Additional file

Additional file 1 **Supplementary figures.** Specific results achieved for the other corpora. (DOCX 368 kb)

## Abbreviations

BOW: bag-of-words
LDA: latent Dirichlet allocation
ATR: automatic term recognition
PICO: the Population, the intervention, comparator and the outcome
SVM: support vector machine
SVD: singular value decomposition
TF-IDF: term frequency/inverse document frequency
ROC: receiver operating characteristic
PRC: precision-recall curve
POS: part-of-speech
RBF: radial basis function
POLY: polynomial
MCMC: Monte Carlo Markov chain
TE: term-enriched
