# Supplementary material for: Supporting systematic reviews using LDA-based document representations
Source: Syst Rev. 2015 Nov 26;4:172. doi: 10.1186/s13643-015-0117-0 (PMC4662004; doi:10.1186/s13643-015-0117-0)
Supplement: Additional file 1 — Supplementary figures. Specific results achieved for the other corpora. (DOCX 368 kb) [file 13643_2015_117_MOESM1_ESM.docx]

# Cooking Skill dataset


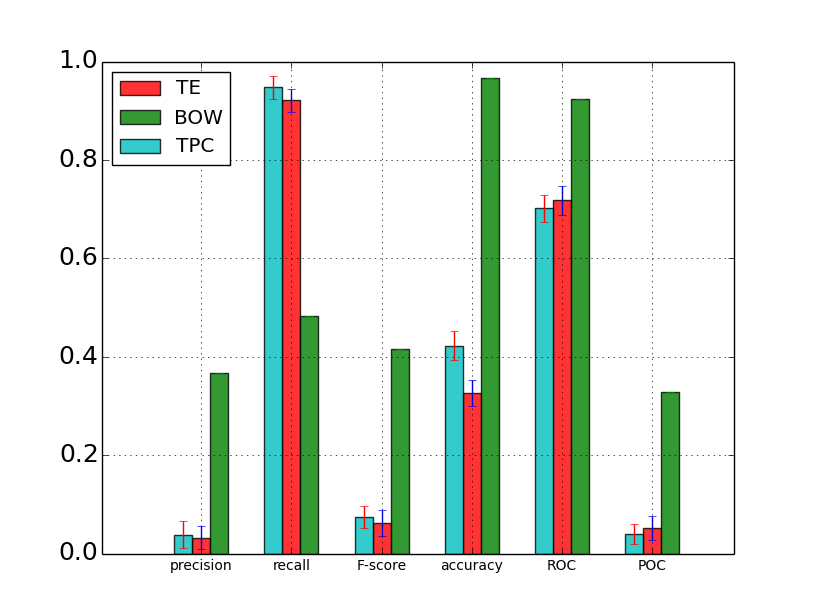


Figure S1 linear kernel function.


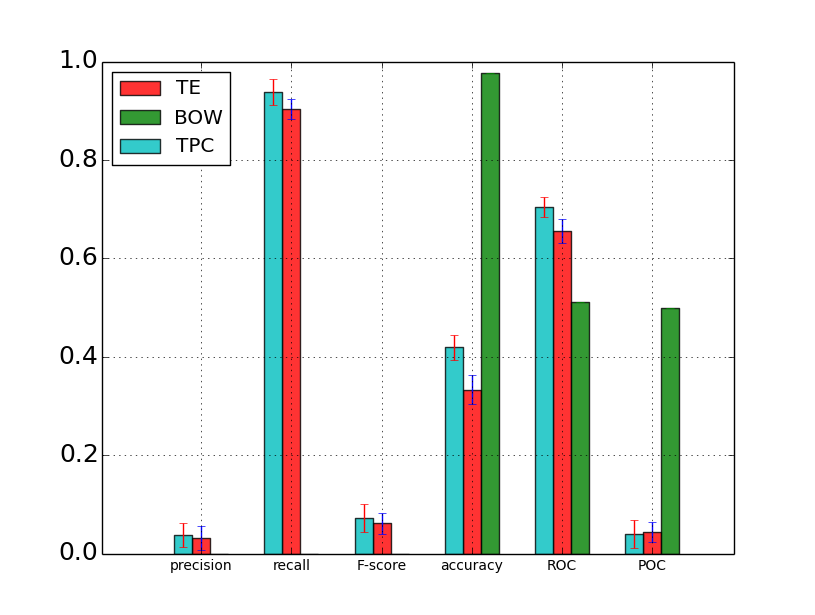


Figure S2 POLY kernel function


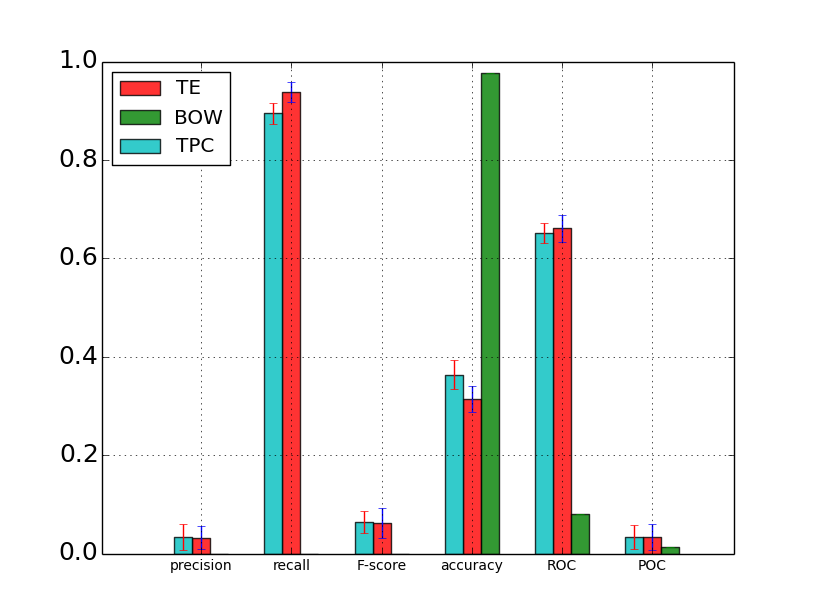


Figure S3 RBF kernel function

# COPD dataset

#
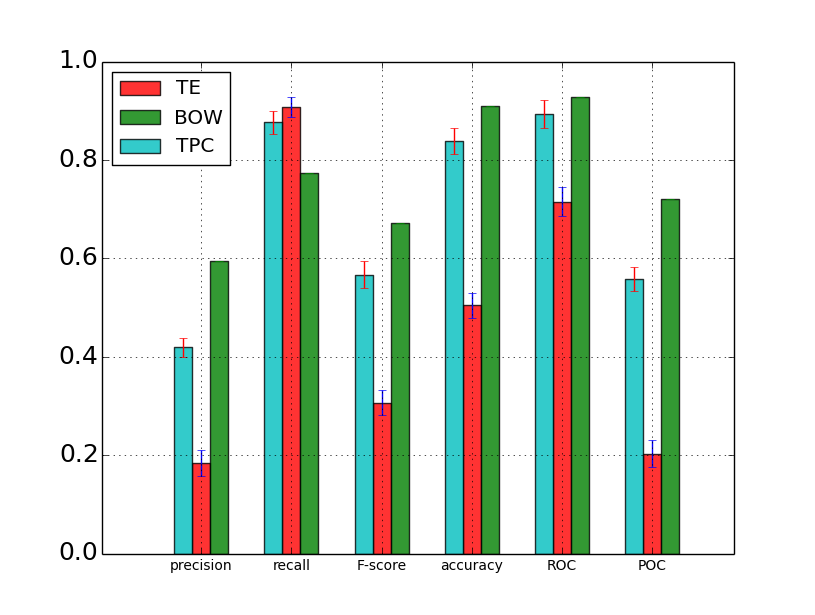


Figure S4 Linear kernel function

#
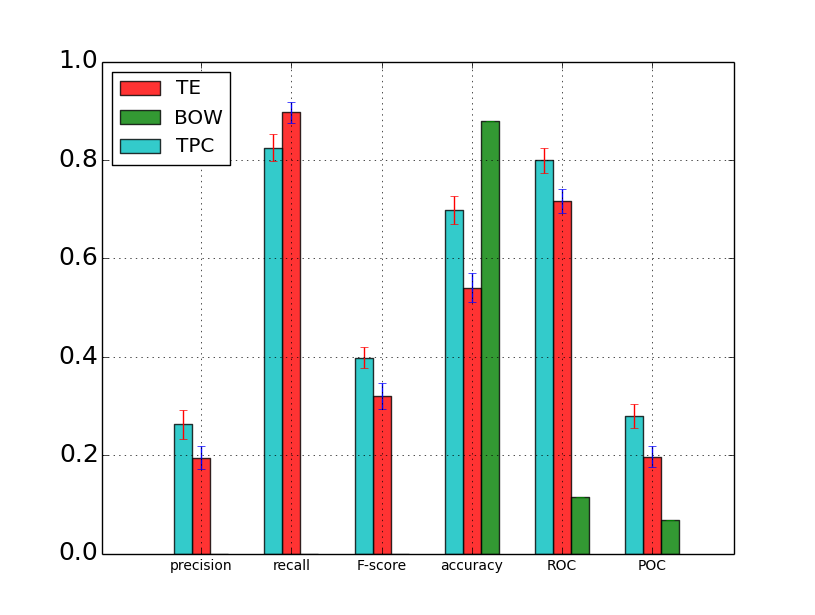


Figure S5 POLY kernel function

#
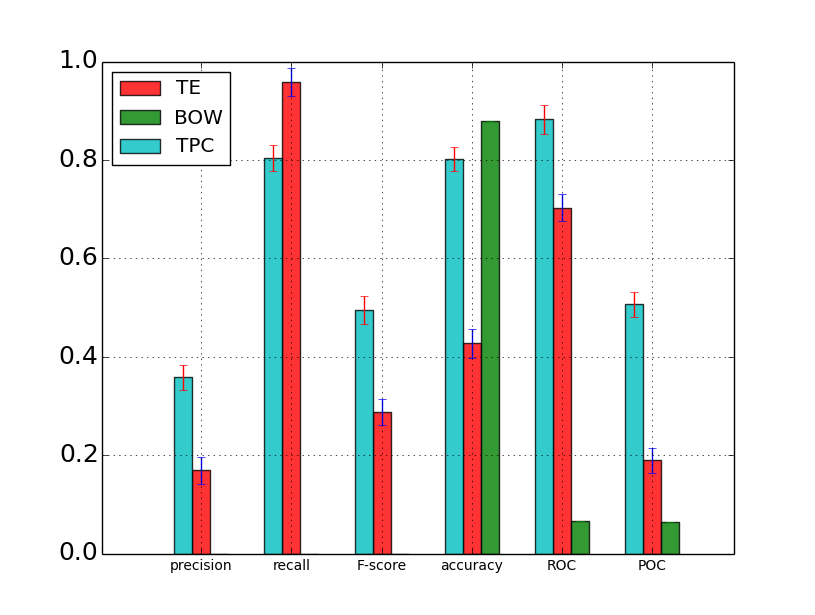


Figure S6 RBF kernel function

# Cigarettes packaging dataset


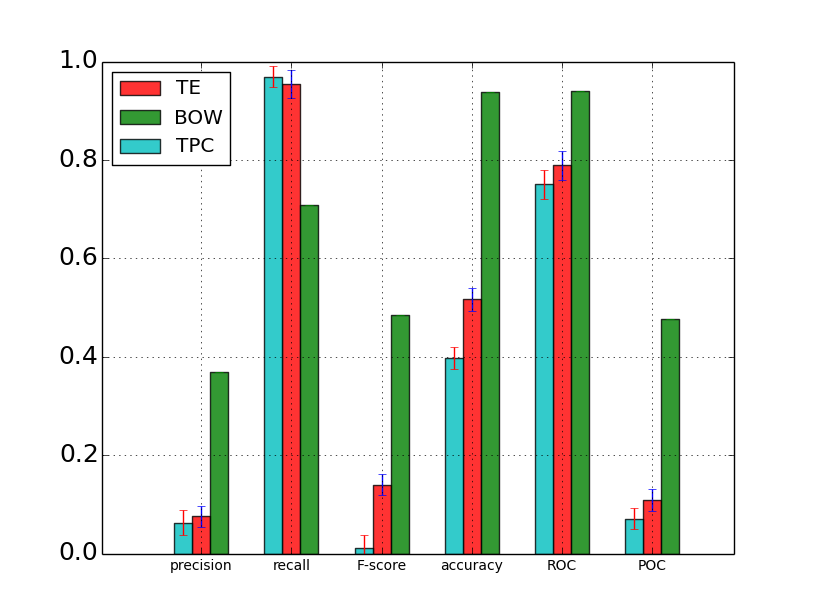


Figure S7 Linear kernel function


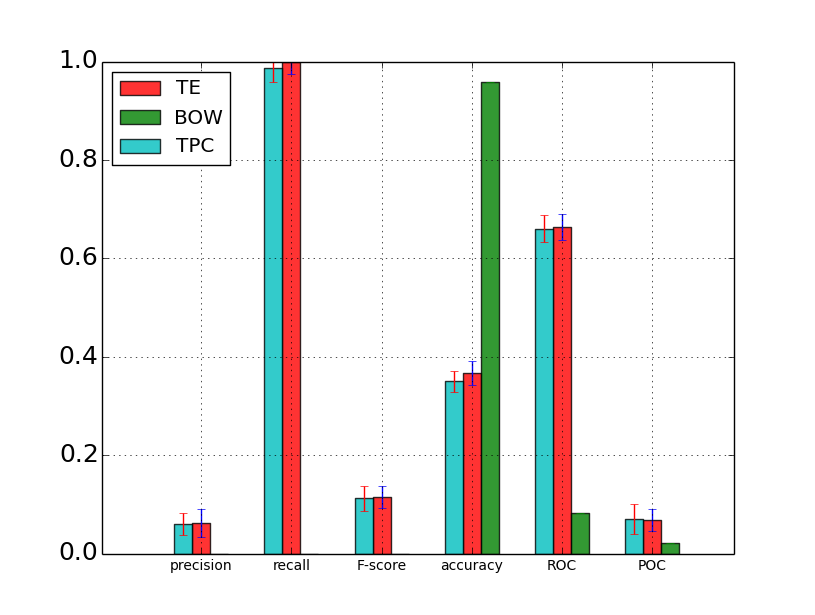


Figure S8 POLY kernel function


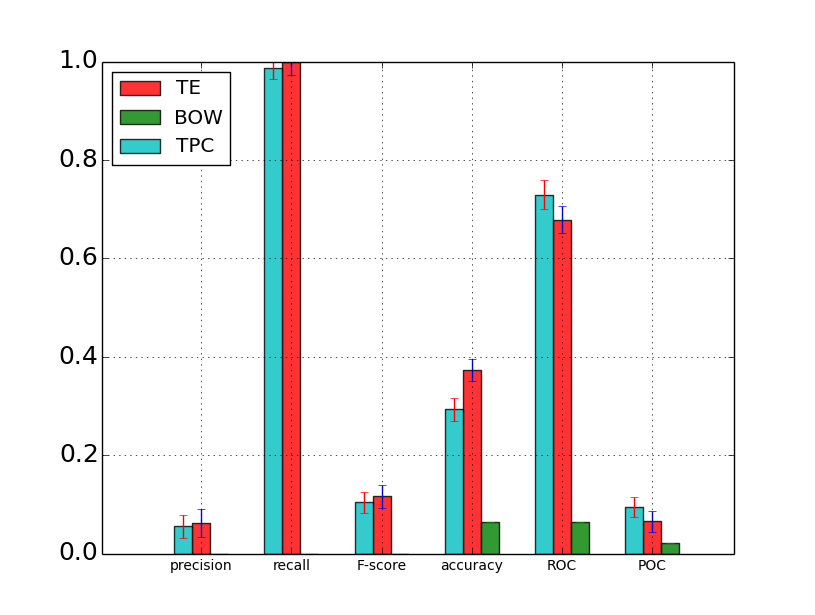


Figure S9 RBF kernel function

# Proton beam dataset


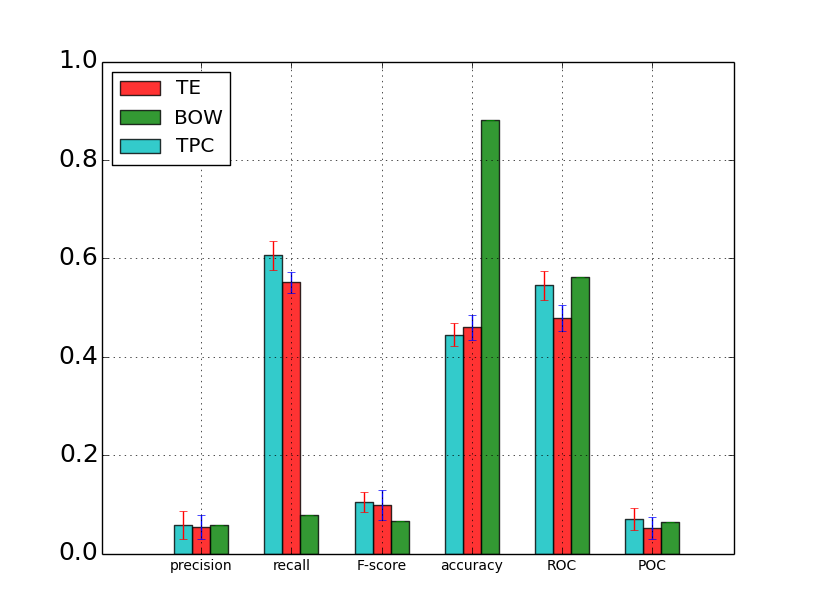


Figure S10 Linear kernel function


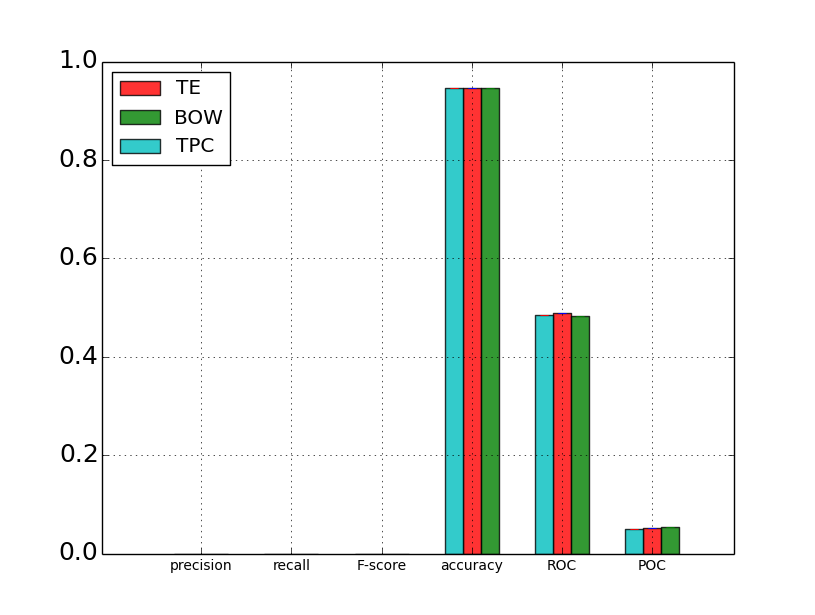


Figure S11 POLY kernel function


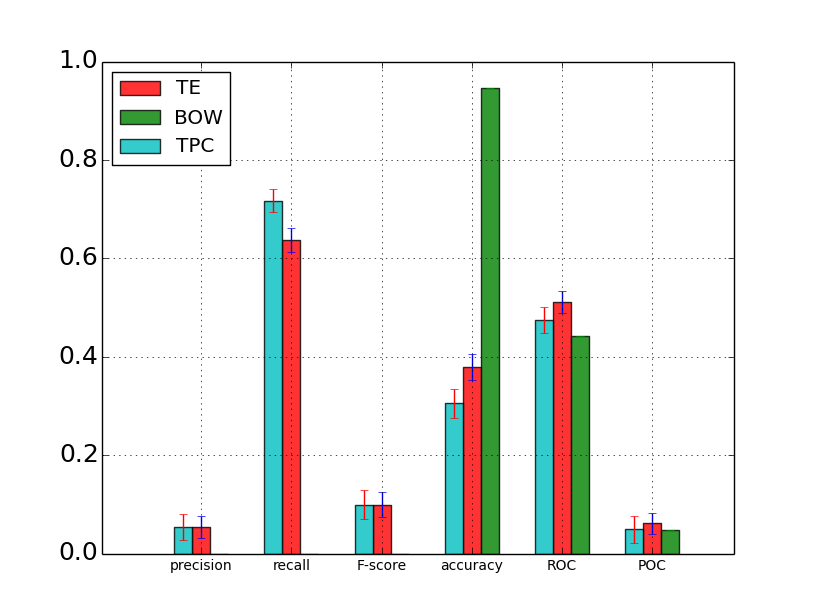


Figure S12 RBF kernel function
